# Composite and Polymeric Materials for Dentistry: Enhancing Antimicrobial and Mechanical Properties

**DOI:** 10.3390/ma16041432

**Published:** 2023-02-08

**Authors:** Grzegorz Chladek

**Affiliations:** Department of Engineering Materials and Biomaterials, Faculty of Mechanical Engineering, Silesian University of Technology, 18a Konarskiego Str., 41-100 Gliwice, Poland; grzegorz.chladek@polsl.pl

Billions of people suffer from dental problems and that number is constantly increasing. Paradoxically, the deteriorating state our teeth is accompanied by the ever-increasing desire to preserve our best facial appearance, which is significantly influenced by dental aesthetics. This favors the dynamic development of dental materials. Special attention has been granted to the possibility of giving materials new or improved properties by the introduction of nano- or submicrometer-size additives, natural oils, new monomers, and other potentially beneficial chemical and structural modifications. Equally important are the new data regarding the colonization of dental materials by pathogenic microbes and their influence on the other properties, as well as the multifactor evaluation of materials recently introduced to the market. Therefore, this Special Issue includes a compilation of different review and research analyses pertaining to the improvement of the antimicrobial and mechanical properties of composite and polymeric materials for dentistry.

The published review articles discuss current knowledge related to selected aspects of the development and function of dental materials. Barszczewska-Rybarek [1] analyzed the relationships between structure and biofunctional properties in the cross-linked dimethacrylate-based matrices used for dental materials. She discussed the influence of the chemical structure, molecular structure represented by the degree of conversion and crosslink density, supramolecular structure related to microgel agglomerate dimensions, and the role of hydrogen bonding in the mechanical properties and water sorption. Kaczmarek et al. [2] presented a review focused on using selected spectroscopic methods for surface analysis of different dental materials, including polymer-based materials. The principles, advantages, limitations, and typical applications of techniques such as Raman spectroscopy, Infrared Spectroscopy, Ultraviolet and Visible Spectroscopy, X-ray spectroscopy, and Mass Spectrometry have been shown. The presented work is of particular interest to dentists conducting research related to materials science. Grazioli et al. [3] summarized current knowledge related to the method of removing adhesives after the debonding of metal orthodontic brackets. Studies on this topic are rare, despite the clinical usefulness. Four different methods of bracket surface preparation were investigated until now: sandblasting, laser, mechanical grinding, and direct flame. All tested methods improved shear bond strength and were clinically acceptable; however, only after using an erbium-doped yttrium aluminum garnet laser were similar values than obtained for new brackets. Nicholson et al. [4] review the experimental methods for the improve mechanical properties of conventional and resin-modified dental cements. Certain fibers (glass, cellulose, basalt) and nanoparticles (TiO_2_, Al_2_O_3_ or ZrO_2_) were indicated as the most promising examples. However, in the case of the nanoparticles, the positive effect was influenced by the morphology of the cement matrix; in particular, the increase in porosity. All described modifications were partially tested only in laboratory investigations, and none have yet seen clinical use. Mishra et al. [5] analyzed literature related to the influence of surface preparation of glass fiber posts on the strength of the bond to dentine. Surface treatment with application of phosphoric acid, hydrogen peroxide, and silane enhances post’s retentiveness. An analysis of the current stage of knowledge regarding the properties of bioinductive materials in direct and indirect pulp capping procedures was presented by Kunert et al. [6]. The authors indicated that calcium silicate cements are characterized by positive properties confirmed by numerous independent research studies; whereas, for a light-cured calcium silicate-based material and a resin-modified glass-ionomer, evidence is insufficient to support the use of these materials in vital pulp therapy.

A widely represented group of research papers was concerned with dental resin-based composites for fillings. Several works related to the development of new materials were presented. The use of dimethacrylates with introduced quaternary ammonium groups is considered as antibacterial dental composites for filling. Chrószcz-Porębska et al. [7] investigated the mechanical properties, as well as the sorption and solubility of experimental matrices composed of six different types of quaternary ammonium urethane-dimethacrylate triethylene glycol dimethacrylate. Despite very good antimicrobial properties, a significant deterioration of mechanical properties and increases in water absorption and solubility were noted. These materials are not attractive in terms of the considered solution, although they may be an interesting proposition in the case of applications requiring lower mechanical properties. Pałka et al. [8] investigated the influence of the addition of liquid rubber (methacrylate-terminated polybutadiene) on the properties of dental composites. The experimental materials presented enhanced shear bond strength values for enamel and dentine, reduced hydrophilicity, and reduced biofilm activity (*Steptococcus mutans*, *Streptococcus sanguinis*); however, they may show cytotoxicity for some formulations. Lapinska et al. [9] investigated the activity of essential oils (rosemary thyme, anise, clove, geranium, cinnamon, limetta, mint, citronella, lavender) against typical oral pathogenic microorganisms (*Streptococcus mutans*, *Lactobacillus acidophilus*, *Candida albicans*) in the context of their incorporation into resin composites. The cinnamon oil was identified as the most promising and introduced in different concentrations into commercially available composite resin. A strong antimicrobial effect of the experimental materials was obtained; however, the authors point out the need to carry out tests of other properties to confirm the favorable characteristics of the materials. Equally important is the research of the materials available on the market. D’Ercole et al. [10] studied the potential to reduce colonization by *Streptococcus mutans* of three commercially available resin-based composites. Investigations have shown that for surfaces prepared in the same way, the materials presented different adherence of bacteria and biofilm accumulation. The authors concluded that the chemical composition of composites will likely play an important role in the process of bacterial adhesion/proliferation; however, they noted the need for further research to confirm their results. Composite materials are complex systems; therefore, it is important for the scientific community to indicate the initial resin formulations for further, more complex experimental analyses. For this reason, Szczesio-Wlodarczyk et al. [11] studied the mechanical properties of resins intended for dental composite matrices containing urethane dimethacrylate (UDMA) and diversified compilations of other dental monomers such as bisphenol A-glycidyl methacrylate (Bis-GMA), triethylene glycol dimethacrylate (TEGDMA), and ethoxylated bisphenol-A dimethacrylate (Bis-EMA). The authors indicated as the most favorable formulations UDMA/Bis-GMA/TEGDMA in proportions of 70/10/20 wt.% and 40/40/20 wt.% and UDMA/Bis-EMA/TEGDMA in proportions of 40/40/20 wt.%, due to their good compilation of flexural strength, flexural modulus, hardness, diametral tensile strength, and water absorption values.

Other important and new dental materials are dental infiltrants used in the treatment of early carious lesions in line with the idea of microinvasive dentistry. Fisher et al. [12] investigated an experimental infiltrant with a chemical composition similar to a commercially available product, but with the addition of a bacteriostatic component (metronidazole). The pilot results suggest that this formulation is not cytotoxic and may be considered as an alternative to the commercial preparation due to its microbilogical action. The developed infiltrant was also investigated with regard to its ability to penetrate into the root cement [13]. Microscopic investigations have shown that the proposed material may be a potentially beneficial solution for the treatment of early carious lesions of the tooth root, as it exhibited a deep penetration into demineralized tissues.

Denture base polymeric materials have been used in dentistry for decades; however, due to their importance and popularity they are still widely investigated by the scientific community. Colonization and penetration of prosthetic materials by *Candida albicans* is a frequently considered problem. Chladek et al. [14] conducted a 90-day experiment with a different methodology to those previously used to verify whether PMMA is penetrated by *Candida albicans* and to investigate its mechanical properties after exposure to yeast-like fungi. Microscopic observations have not confirmed the penetration of fungi into the material. A decrease in surface hardness was registered, while flexural strength, flexural modulus, tensile strength, impact strength, and ball indentation hardness were at the same level as controlNumerous yeast cells were observed on the surface in crystalized structures and in traces after grinding, suggesting that not penetration, but the deterioration of surface quality may create microareas that are difficult to disinfect in clinical conditions. Other investigations related to the problem of PMMA colonization by *Candida albicans* were presented by Petrovi et al. [15]. They introduced 3 to 12% of oleic acid and registered a decrease in the water contact angle and metabolic activity of yeast cells. Investigations of other biofunctional properties are still needed. An important direction of research into denture base resins is the development of materials with antimicrobial properties. Strong antifungal properties were noted after incorporation of submicrometer inorganic particles of silver sodium hydrogen zirconium phosphate. Modification results in reduced flexural strength, impact strength, and enhanced solubility; however, obtained values were at acceptable levels. On the other hand, favorable changes were also recorded; hardness and flexural modulus increased whereas volume loss during the wear test decreased with the introduction of ceramic particles [16]. Chuchulska et al. [17], in the technological study, tested how the small changes in the laboratory protocol of polyamide prosthetic base materials may influence the surface texture. Investigations have shown that modifying the process by altering the melting temperature by 5 °C and the pressure by 0.5 Bar during injection molding can reduce roughness for some materials.

Other materials, which are extremely important from a practical point of view, have been tested to determine or predict their clinical properties. Sfondrini et al. [18] study the effect of tooth brushing on surface wear and the mechanical properties of a glass fiber-reinforced composite orthodontic retainer during an in vitro experiment. The stainless-steel wires, flowable resin composite covered, and spot-bonded fiber reinforced composites were tested after 26 min and 60 min of tooth brushing. The three-point bending test and SEM investigations showed a significant reduction of flexural strength and signs of wear on both fiber-reinforced materials. The authors suggest that proposed solutions need further tests before routine application in clinical practice. Rini Behera et al. [19] presented a one-year in vivo comparison of lithium disilicate and zirconium dioxide class II inlay restorations. The survival rate was evaluated, and one failure was observed in the zirconium dioxide group (the survival probability was 93%) when no failure was observed in the lithium disilicate group. Both types of inlays presented comparable surface roughness, marginal adaptation, anatomic form, occlusal contact, and proximal contact during the experiment, but the color and translucency match was far worse for the zirconium dioxide restorations. Szczesio-Wlodarczyk et al. [20] investigated the effect of different liquids (ethanol, soda solution, and green tea) on the mechanical properties of dental cements used for cementing restorations. The ethanol solution showed the greatest influence on the hardness of composite cement when soda solution was applied to zinc-polycarboxylate cement. Diametral tensile strength values were unchanged for composite cements, increased after exposure of zinc-polycarboxylate cement to ethanol, and decreased in soda solution for glass-ionomer cement. In vitro experiments conducted by Sobolewska et al. [21] demonstrated a slight-to-moderate toxic effect on human pulp fibroblasts of denture adhesive creams, but the authors suggest the need for in vivo studies to verify these results in patients. Łysik et al. [22] investigated the degradation of polylactide (PLA) and polycaprolactone (PCL) as a consequence of biofilm presence (*Candida krusei* and *Steptococcus mutans*) in the artificial saliva. After 56 days, their experiment has shown that as a consequence of the microorganisms, the surface morphologies were changed and molecular weight and mechanical properties decreased.

Special attention should be paid to the newly developed materials with bioactive properties, which are expected to find application in dentistry, implantology, and dental surgery. Babu et al. [23] conducted an experiment with zirconium-doped calcium phosphate-based bioglasses, which should be considered as a prominent solution. A bioglass system was synthesized by the melt quenching process. The results shown that increases in zirconia concentration increase the glass-forming ability and thermal stability of materials. Spectroscopic investigations confirmed the presence of a thin hydroxyapatite layer on the sample surface after incubation in SBF solution. The process of glass degradation after incubation in SBF increased with time but decreased with the increase of ZrO_2_ concentration. The results confirmed the suitability of bioglasses tested for bone-related applications.

The quest editor wishes to give special thanks to all the authors, and to the editorial team of *Materials* for the collaborative peer review and publishing process. I hope that readers will enjoy the Special Issue “Composite and Polymeric Materials for Dentistry: Enhancing Antimicrobial and Mechanical Properties” and will find within its pages new knowledge and ample inspiration for future research.
